# Beneficial Regulatory Effects of Polymethoxyflavone—Rich Fraction from Ougan (*Citrus reticulata* cv. *Suavissima*) Fruit on Gut Microbiota and Identification of Its Intestinal Metabolites in Mice

**DOI:** 10.3390/antiox9090831

**Published:** 2020-09-06

**Authors:** Jiebiao Chen, Yue Wang, Tailin Zhu, Sijia Yang, Jinping Cao, Xian Li, Li-Shu Wang, Chongde Sun

**Affiliations:** 1 Laboratory of Fruit Quality Biology/Zhejiang Provincial Key Laboratory of Horticultural Plant Integrative Biology/The State Agriculture Ministry Laboratory of Horticultural Plant Growth, Development and Quality Improvement, Zhejiang University, Zijingang Campus, Hangzhou 310058, China; jiebiaochen@zju.edu.cn (J.C.); fruit@zju.edu.cn (Y.W.); flannery@zju.edu.cn (T.Z.); 3170100347@zju.edu.cn (S.Y.); caojinpingabc@126.com (J.C.); xianli@zju.edu.cn (X.L.); 2 Division of Hematology and Oncology, Department of Medicine, Medical College of Wisconsin, Milwaukee, WI 53226, USA; liswang@mcw.edu

**Keywords:** Ougan (*Citrus reticulata* cv. *Suavissima*), polymethoxyflavones, gut microbiota, metabolism in vivo, metabolite identification, beneficial regulatory effect

## Abstract

Polymethoxyflavones (PMFs) are special flavonoids in citrus fruits that have been suggested to be beneficial to human health. However, whether PMFs in citrus fruit alter human gut microbiota is not well understood. The aim of the present study was to investigate the effects of PMF-rich fraction from Ougan (*Citrus reticulata* cv. *Suavissima*) on gut microbiota and evaluate the intestinal metabolic profile of PMFs in Institute of Cancer Research mice. The main components of the PMF-rich fraction were nobiletin, tangeretin, and 5-demethylnobiletin. The composition of the gut microbiota was analyzed using 16S ribosomal DNA sequencing. The results showed that after oral administration, the composition of mice gut microbiota was significantly altered. The relative abundance of two probiotics, *Lactobacillus* and *Bifidobacterium*, were found to increase significantly. A total of 21 metabolites of PMFs were detected in mice intestinal content by high performance liquid chromatography electrospray ionization tandem mass spectrometry, and they were generated through demethylation, demethoxylation, hydroxylation, and glucuronidation. Our results provided evidence that PMFs have potential beneficial regulatory effects on gut microbiota that in turn metabolize PMFs, which warrants further investigation in human clinical trials.

## 1. Introduction

The gut microbiota is a complex ecosystem inhabiting in the gastrointestinal tract and consisting of a diverse microbiotic community living in symbiosis with the host [1]. Mounting studies have suggested that the gut microbiota possesses many vital functions, functioning as an indispensable part of the human body to maintain our health, including regulating the energy metabolism, immune function, and hormonal balance [2,3,4]. The gut microbiota can directly shape our health states. A critical part of gut immune system is the significant role it plays in stabilizing the host defense by protecting against pathogens, and there will be a risk of immune system-mediated diseases if it is out of balance [4]. The microbial compositions and abundance are directly and indirectly changed by the dietary patterns, antibiotics, probiotics, and lifestyle of the individual, among which the diet is considered the primary modulator of the gut microbiota [2]. This is not surprising because distinct food components selectively enrich microorganisms that are able to utilize these nutrients and support microbial metabolic cross-feeding, leading to the maintenance of a diverse and balanced community [4].

Fruit is an important component of the human daily diet that is abundant in phenolic compounds. As an extensively distributed kind of phytochemical, it is well accepted that phenolic compounds possess many bioactivities [5]. Recent studies have reported that some fruits such as cranberry [6], pomegranate [7], blackcurrant [8], apple, grape [9], and blueberry [10] are able to alter the gut microbiota, either by increasing the abundance of beneficial bacteria or reducing the abundance of harmful bacteria. Moreover, the gut microbiota regulation abilities are believed to be attributed to the abundant phenolic compounds in these fruits [11]. Flavonoids are naturally occurring phenolic compounds that can be divided into six classes according to their molecular structure: flavones, flavanones, flavonols, isoflavones, anthocyanidins, and flavanols [12]. Most plant-derived flavonoids are present in a glycoside form and are conjugated with sugars. Due to their hydrophilicity, they are not easily absorbed and metabolized by the upper gastrointestinal tract. Evidence has shown that after oral administration, a large fraction of flavanone reaches the colon intact, where it is metabolized into various small absorbable phenolics by the gut microbiota [13]. The gut microbiota also play an important role in metabolizing the flavonoids that cannot be metabolized by human enzymatic hydrolysis, such as *C*-glycosyl flavonoids [14] and rhamno-glucosides [13].

Citrus fruits are rich sources of flavonoids, and those specifically derived from citrus fruits are called citrus flavonoids. Among the flavonoids, flavanones are the highest in content [15], while the content of polymethoxyflavones (PMFs) varies among different types and tissues of citrus fruit [16]. Studies have been carried out to investigate the interaction between the intestinal metabolism of citrus flavanones and gut microbiota. For example, the representative citrus flavanone glycosides hesperidin and naringin are mainly metabolized by intestinal bacteria, resulting in the generation of their aglycone forms and other smaller phenolics. As a result, these citrus flavanones and their metabolites influence the composition and activity of the gut microbiota [17]. However, only a few studies have investigated the bacterial metabolism of PMFs and the influence of PMFs on the composition of gut microbiota.

PMFs are flavones that contain four or more methoxy groups (OCH_3_) on their basic benzo-γ-pyrone (15-carbon, C6-C3-C6) skeleton with a carbonyl group at the C4 position. In general, PMFs exclusively exist in citrus fruits, and they are principally identified in the flavedo of certain categories. The major PMFs distributed in citrus fruit are nobiletin and tangeretin [18]. The research community has been interested in PMFs for many years because of its broad spectrum of bioactivities. Plenty of studies have indicated that PMFs show potent protective effects on humans’ physical health, such as anti-inflammation [19], anticancer effects [20], neuroprotection [21], and metabolic disorder regulating functions [22]. Many properties are partly related by their antioxidant bioactivity, and there is a growing interest in the usage of citrus PMFs as a strategy to prevent oxidative damage in various health disorders [23]. Bioavailability is a crucial factor that determines the biological activity of a certain substance. Experiments in vivo and in vitro have been carried out to study the absorption and metabolism of PMFs. The metabolites of PMFs closely associate with their bioactivities, and a growing body of evidence has suggested that their metabolites may have greater activity compared to parental compounds [24]. PMFs undergo a series of complex biotransformations in vivo, and there is evidence that the gut microbiota plays a significant role in this process [25].

In this study, PMF-rich fraction was extracted from Ougan (*Citrus reticulata* cv. *Suavissima*), a characteristic citrus variety in Zhejiang province, which is abundant in PMFs according to our previous findings [16]. The main objective of the present study was to study the effects of PMFs from Ougan on the composition of the gut microbiota, as well as how PMFs are converted in the gastrointestinal tract of mice.

## 2. Materials and Methods

### 2.1. Materials and Animals

Ougan fruits at commercial maturity were collected in December 2017, from Lishui city, Zhejiang province, China. Fruits without mechanical damage, diseases, or pests were selected and stored at 4 °C for further experiments.

Four-week-old male Institute of Cancer Research (ICR) mice (*n* = 70) were bred with four animals per cage in the Laboratory Animal Center of Zhejiang University. ICR mice are a general-purpose model that are used in particular in toxicology, neurobiology, oncology, epidemiology, infection, and pharmacology testing, as well as in product safety testing. The mice were housed in a controlled environment (temperature controlled at 23 ± 3 °C, 12 h daylight cycle) with food and water ad libitum. All the protocols in this study were approved by the Committee on the Ethics of Animal Experiments of Zhejiang University (permission number: ZJU20200045). The mouse feed was purchased from Fbsh Bio-Pharmaceutical Co., Ltd. (Shanghai, China), and was flavonoid-free.

### 2.2. Extraction of PMF-Rich Fraction from Ougan Fruit

The extraction of PMF-rich fraction was performed according to our previous study [26] with modifications. After washing, the peel of Ougan fruit was separated and collected. A total of 100 g of peel was accurately weighed and grinded in 500 mL of 80% ethanol using a juice extractor. Grinded peel was then ultrasonically extracted with a frequency of 53 kHz for 1 h. Four-layer gauze and filter paper were subsequently used for filtering, and the filtrate was dried in a vacuum rotatory evaporator at 37 °C. After that, the residue was dissolved in double-distilled H_2_O (ddH_2_O). Sep-pak C18 cartridge columns (20 cc, 5 g sorbent, Waters Corp., Milford, MA, USA) were used for enrichment of the PMF fraction. After the samples were loaded, ddH_2_O of 20 bed volume (BV) was eluted to remove the organic acids and sugars. Then, the flavonoid components with high polarities were removed by 8 BV 30% and 1 BV 35% aqueous methanol solution successively. Next, the PMF-rich fraction was eluted by 1 BV 100% methanol. The eluent was vacuum-dried for further experiments.

### 2.3. Collection of the Intestinal Contents

After 1 week of acclimation, 60 mice were administrated with PMF-rich fraction at a dose of 200 mg·kg^−1^ BW·d^−1^ by gavage. The contents in the small intestine, cecum, and colon were collected and combined 1 h, 2 h, 3 h, 6 h, 12 h, and 24 h after the PMF administration, with 10 mice at each time point. The controls were administrated with equal volume of pure water (*n* = 10), and the intestinal contents were collected 1 h after gavage. All the intestinal contents were snap frozen in liquid nitrogen and then kept at −80 °C for subsequent experiments.

### 2.4. Extraction of Flavonoid Components from the Intestinal Contents

The intestinal contents from five mice of each group were randomly selected for the extraction of flavonoid components. For each sample, 0.2 g of intestinal contents was accurately weighed and ultrasonically extracted with 2 mL of 80% ethanol, with a frequency of 53 kHz for 30 min. The mixture was then centrifuged at 5000 rpm for 10 min at room temperature, and the supernatant was collected. The extraction steps were repeated three times, and the supernatant was combined and dried in a vacuum rotatory evaporator at a temperature of 37 °C. The residue was dissolved in 3 mL of ddH_2_O. Sep-pak C18 cartridge columns (1 cc, 0.1 g sorbent, Waters Corp., Milford, MA, USA) were used for enrichment of the flavonoid components. After the samples were loaded, we eluted ddH_2_O of 20 BV to remove the non-flavonoid fraction. Then, the flavonoid components were eluted by 2 BV 100% methanol. Next, 200 μL eluent of each sample was pipetted and combined. The mixed solution was vacuum dried at 37 °C, and the residue was dissolved in 200 μL of HPLC-grade methanol for further analysis. Likewise, 200 μL eluent of each control group sample was mixed and vacuum-dried, and then dissolved in 200 μL of HPLC-grade methanol as the background control.

### 2.5. Identification of Flavonoids

High performance liquid chromatography electrospray ionization tandem mass spectrometry (HPLC–ESI–MS/MS) was performed to identify the flavonoid components in PMF-rich fraction and intestinal content. Two mixed samples were used for the identification of intestinal metabolites. Detailed conditions were described in our previous publication with some modifications [16].

HPLC was performed on a SunFire C18 (5 μm, 4.6 × 250 mm) column. The linear gradient elution was performed using a mobile phase composed of mixture of solution A (water containing 0.1% formic acid) and B (HPLC grade acetonitrile). The optimized condition is listed as follows: solution B: 0–5 min, 20%; 5–10 min, 20–27%; 10–15 min, 27%; 15–25 min, 27–40%; 25–35 min, 40–60%; 35–40 min, 60–80%; 40–42 min, 80–100%; 42–45 min, 100–20%; 45–50 min, 20%. Detection temperature was 25 °C, injection volume was 10 μL, the flow rate was 1 mL/min, and the compounds were detected between 200 and 500 nm.

Mass spectrometric analysis was operated on a AB Triple TOF 5600^plus^ system. Briefly, the nebulizer pressure was set to 45 psi, and the drying gas flow rate was 5 L/min. The flow rate and the temperature of the sheath gas were 11 L/min and 350 °C, respectively. All the produced ions were introduced into the TOF–MS instrument for accurate mass determination.

### 2.6. DNA Extraction and 16S ribosomal DNA Sequencing of Intestinal Content from Mice

The intestinal contents from the remaining five mice of each group were used for 16S ribosomal DNA sequencing to study the composition of the gut microbiota. The DNA of the intestinal content was extracted using MagPure Stool DNA KF kit B (Magen, Hong Kong, China) following the manufacturer’s instructions. The extracted DNA was quantified with a Qubit Fluorometer, and the V3–V4 hypervariable regions of the bacteria 16S ribosomal DNA gene were amplified by PCR with barcode-indexed primers 515F (5′-GTGCCAGCMGCCGCGGTAA-3′) and 806R (5′-GGACTACHVGGGTWTCTAAT-3′). The validated libraries were used for sequencing on the Illumina HiSeq 2500 platform (BGI, Shenzhen, China). Detailed PCR conditions are listed in Table S1.

### 2.7. Sequencing Data Processing

After the raw reads were filtered, we added paired-end reads to tags by FLASH (v1.2.11) to obtain the tags. The tags were clustered into operational taxonomic units (OTUs) with a cutoff value of 97%, then out-representative sequences were taxonomically classified using RDP Classifier v.2.2 with a minimum confidence threshold of 0.6, and trained on the Greengenes database v201305 by QIIME v1.8.0. The USEARCH_global was used to compare all tags back to OTU to obtain the OTU abundance statistic table of each sample.

### 2.8. Statistical Analysis

The HPLC–ESI–MS/MS data was analyzed by PeakView software (version 1.2, AB SCIEX, Toronto, ON, Canada).

Significant species were determined by R (v3.4.1) on the basis of the Wilcoxon test or Kruskal–Wallis test. All the results were considered statistically significant at *p* < 0.05. Alpha and beta diversity were estimated by MOTHUR (v1.31.2) and QIIME (v1.8.0) at the OTU level. Principal component analysis (PCA) in OTUs was plotted with R package “ade4”. Sample cluster was conducted by QIIME (v1.8.0) on the basis of UPGMA. The heat map of different classification levels was plotted with R package “gplots”.

## 3. Results

### 3.1. Identification of PMF-Rich Fraction

**Figure 1:**
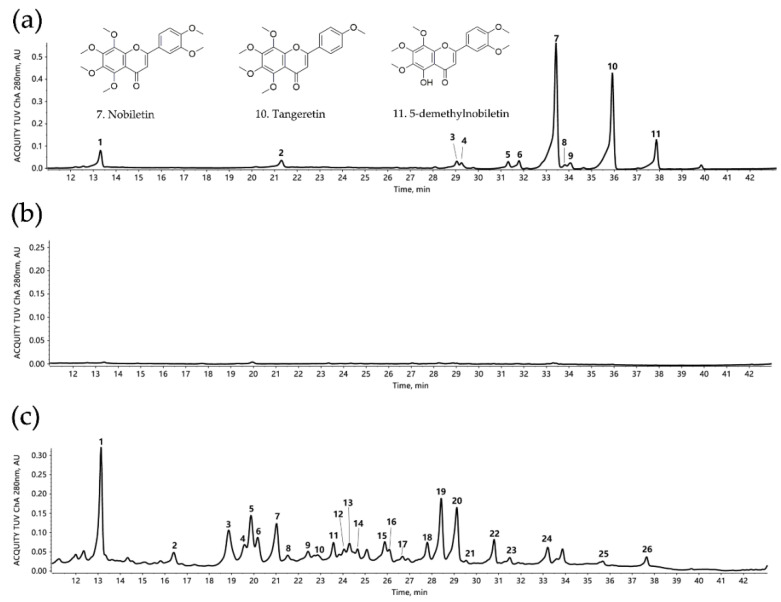
HPLC chromatogram of Ougan PMF-rich fraction and mice intestinal content samples (λ = 280nm). (**a**) HPLC chromatogram of Ougan PMF-rich fraction. 1: neohesperidin; 2: poncirin; 3: isosinensetin; 4: monohydroxy-pentamethoxyflavone; 5: sinensetin; 6: tetramethyl-*O*-isoscutellarein; 7: nobiletin; 8: 5,4′-dihydroxyl-3,7,8,3′-tetramethoxyflavone; 9: tetramethyl-*O*-scutellarein; 10: tangeretin; 11: 5-demethylnobiletin. Nobiletin (7), tangeretin (10), and 5-demethylnobiletin (11) are the three major components in Ougan PMF-rich fraction. (**b**) HPLC chromatogram of intestinal content of the mixture of control group. (**c**) HPLC chromatogram of intestinal content of the mixture of all groups. 1: neohesperidin; 2: nobiletin-*O*-glucuronide (1); 3: tangeretin-*O*-glucuronide (1); 4: nobiletin-*O*-glucuronide (2); 5: nobiletin-*O*-glucuronide (3); 6: dihydroxy-tetramethoxyflavone (1); 7: dihydroxy-monomethoxyflavone; 8: dihydroxy-trimethoxyflavone; 9: dihydroxy-tetramethoxyflavone (2); 10: monohydroxy-trimethoxyflavone; 11: tangeretin-*O*-glucuronide (2); 12: dihydroxy-tetramethoxyflavone (3); 13: dihydroxy-tetramethoxyflavone (4); 14: nobiletin-*O*-glucuronide (4); 15: monohydroxy-pentamethoxyflavone (1); 16: trihydroxy-flavone; 17: monohydroxy-tetramethoxyflavone (1); 18: monohydroxy-pentamethoxyflavone (2); 19: monohydroxy-tetramethoxyflavone (2); 20: monohydroxy-pentamethoxyflavone (3); 21: dihydroxy-dimethoxyflavone; 22: monohydroxy-tetramethoxyflavone (3); 23: tetramethoxyflavone; 24: nobiletin; 25: tangeretin; 26: 5-demethylnobiletin.

A total of 11 flavonoid compounds were identified in PMF-rich fraction (Table 1 and Figure 1a), including two flavanone-*O*-glycosides (neohesperidin and poncirin) and nine PMFs. The top three components in this fraction were nobiletin, tangeretin, and 5-demethylnobiletin, and they were in proportions of 48.85%, 31.16%, and 5.05% respectively; the three combined was 85.06%. We previously reported that after C18 Solid Phase Extraction (SPE) enrichment, the proportion of combined nobiletin, tangeretin, and 5-demethylnobiletin increased from 58.7% to 85.3% [26]. Thus the result from this current study was consistent with our previous finding.

Interestingly, isomerization was frequently found among these substances, for example, sinensetin, isosinensetin and tangeretin, tetramethyl-*O*-scutellarein and tetramethyl-*O*-isoscutellarein, monohydroxy-pentamethoxyflavone, and 5-demethylnobiletin.

### 3.2. Identification of Metabolites of PMFs in Intestinal Content

The HPLC profile of the mixture of samples from the control group is shown in Figure 1b, which served as a background control, with the HPLC profile of the mixture of all samples that contained control and PMF-treated groups being displayed in Figure 1c. Compared with Figure 1c, the chromatogram in Figure 1b is much smoother and steadier, which indicates that the flavonoids in the intestinal content of control group were in an undetectable level. Moreover, the difference between these two figures suggested that the administration of PMF-rich fraction caused a dramatic change in intestinal content by producing many flavonoid metabolites.

We determined 26 compounds using HPLC–ESI–MS/MS (Figure 1c and Table 2). Among the 26 detected compounds, 5 of them were identified to be parental compounds of PMF-rich fraction (neohesperidin, monohydroxy-pentamethoxyflavone, nobiletin, tangeretin, and 5-demethylnobiletin), which suggested that PMFs had not been completely metabolized 1 h after they were consumed. Further, 21 compounds were tentatively predicted to be the metabolites of the PMF-rich fraction. These metabolites are mainly generated through demethylation, demethoxylation, hydroxylation, and glucuronidation. Metabolites varied with the number and position of chemical groups participating in demethylation, demethoxylation, and hydroxylation, including monohydroxy-trimethoxyflavone, monohydroxy-tetramethoxyflavone, monohydroxy-pentamethoxyflavone, dihydroxy-monomethoxyflavone, dihydroxy-dimethoxyflavone, dihydroxy-trimethoxyflavone, dihydroxy-tetramethoxyflavone, trihydroxy-flavone, and tetramethoxyflavone. Further, glucuronidation was found to occur after the demethylation of nobiletin and tangeretin, generating corresponding conjugates.

Isomerization was widely found among the metabolites. Specifically, two monohydroxy-tetramethoxyflavones, two monohydroxy-pentamethoxyflavones, four dihydroxy-tetramethoxyflavones, four nobiletin-*O*-glucuronides, and two tangeretin-*O*-glucuronides were detected in the intestinal content. Therefore, this finding suggested that the reaction position was of great diversity. The co-existence of multiple isomers created hurdles for accurately determining the structure of some metabolites. Moreover, it should be noted that the structure of the three main parental compounds (nobiletin, tangeretin, and 5-demethylnobiletin) was of high similarity, which caused barriers to match the metabolites with their corresponding parental compounds. In addition, there were also possibilities that some metabolites were generated through a series of reactions successively. Thus, further study was required to dissect the metabolic pathway of PMFs.

### 3.3. Effects of PMF-Rich Fraction on Gut Microbiota

**Figure 2:**
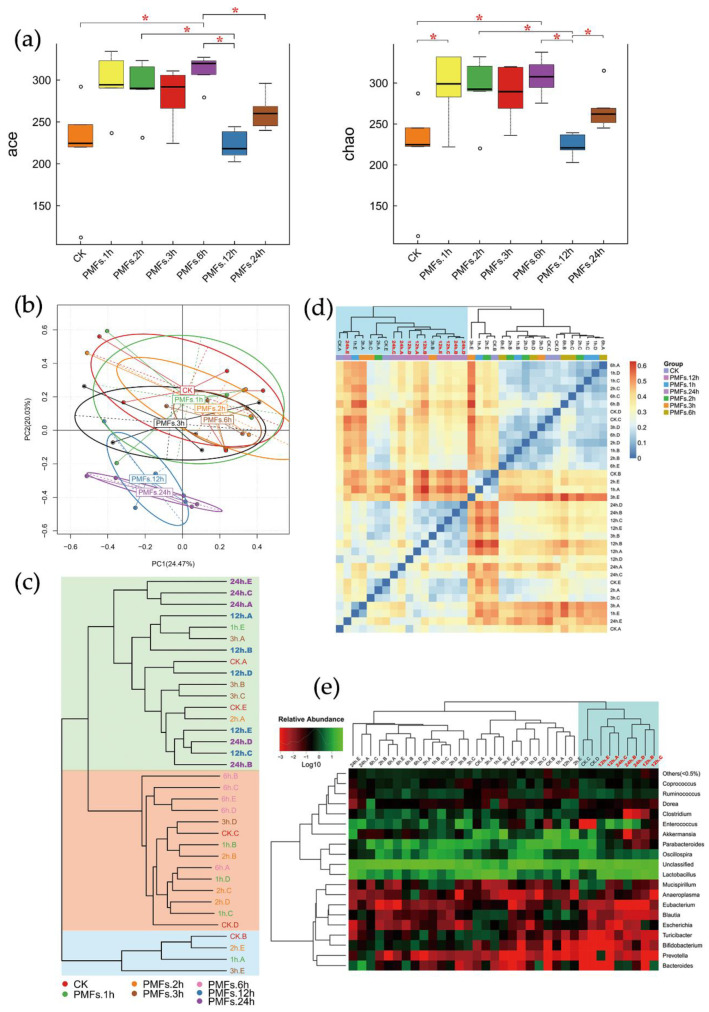
Administration of PMF-rich fraction significantly altered the composition of mice gut microbiota. (**a**) Alpha diversity indices boxplot among groups. Five lines from bottom to top is the minimum value, the first quartile, median, the third quartile, and the maximum value, and the abnormal values are outliers shown as “o”; * *p* < 0.05. (**b**) Principal component analysis (PCA) based on operational taxonomic unit (OTU) abundance. *x*-axis, first principal component, and *y*-axis, second principal component. Numbers in brackets represent contributions of principal components to differences among samples. A dot represents each sample, and different colors represent different groups. (**c**) Samples clustering (weighted unifrac). The word with the same color represents the samples in the same group, the shorter distance between samples represents high similarity. Samples in the same background color represent a high similarity. (**d**) Beta diversity heat map (weighted unifrac). Weighted unifrac value was used to measure beta diversity. Groups PMFs.12h and PMFs.24 h were clustered into the same group highlighted in blue in the top-left corner. (**e**) Log-scaled percentage heat map based on the relative abundance of each species in each sample (genus level). Longitudinal clustering indicates the similarity of all species among different samples, and the horizontal clustering indicates the similarity of certain species among different samples. The closer the distance, the shorter the branch length, and the more similar the species composition between the samples. Relative abundance values were all log-transformed. Most of the samples of groups PMFs.12h and PMFs.24h were clustered into the same group, highlighted in blue in the top-right corner.

We obtained a total of 2,137,084 high-quality 16S ribosomal DNA gene sequence tags from intestinal contents from 35 mice with an average read length of 252 bp (SD = 1 bp). Filtered tags were clustered into OTU by using the Greengene database at 97% similarity. Alpha diversity of each sample was analyzed and the results are displayed in Figure 2a and Figure S1. The diversity of species of the gut microbial community reflected by Shannon and Simpson indexes showed no significant difference among seven groups (*p* > 0.05, *p* (Shannon) = 0.20873, *p* (Simpson) = 0.51176), whereas the species richness indicated by Ace and Chao indexes was significantly increased in the PMF groups compared with the control group (*p* < 0.05, *p* (Ace) = 0.00671, *p* (Chao) = 0.01441), except the PMF 12h group.

Principle component analysis (PCA) was performed to show the differences of microbial composition among different groups at the OTU level. As depicted in Figure 2b, samples from groups PMFs.12h and PMFs.24h formed a cluster that was different from metagenomes derived from clusters formed by the remaining five groups. Therefore, administration of PMF-rich fraction led to the alterations of the gut microbial community 12–24 h after it was consumed.

Similarity in composition of species among samples was evaluated. The clustering results were shown in Figure 2c. As shown in this figure, groups PMFs.12h and PMFs.24h were grouped into one cluster (with a green background), and the other five groups formed the other cluster. This suggested that the gut microbial compositions between groups PMFs.12h and PMFs.24h were similar, and they were distinguished from the control group and other early time points.

Similarly, we carried out heat map analysis on the basis of the relative abundance of each species at the genus and species levels. The distance algorithm was “Euclidean”, and the clustering method was “complete”. As displayed in Figure 2e and Figure S2, at the genus and species levels, most of the samples of group PMFs.12h and group PMFs.24h were clustered into the same cluster, which indicated that the microbial compositions of these two groups have high similarity.

We further performed analysis of beta diversity to evaluate the difference of samples in the complexity of species. The distance algorithm was “weighted unifrac”. The heat map of beta diversity distribution is shown in Figure 2d. Groups PMFs.12h and PMFs. 24h were clustered into the same cluster, suggesting a high similarity in their microbial compositions, which was consistent with the results of PCA, clustering analysis, and heat map analysis.

**Figure 3:**
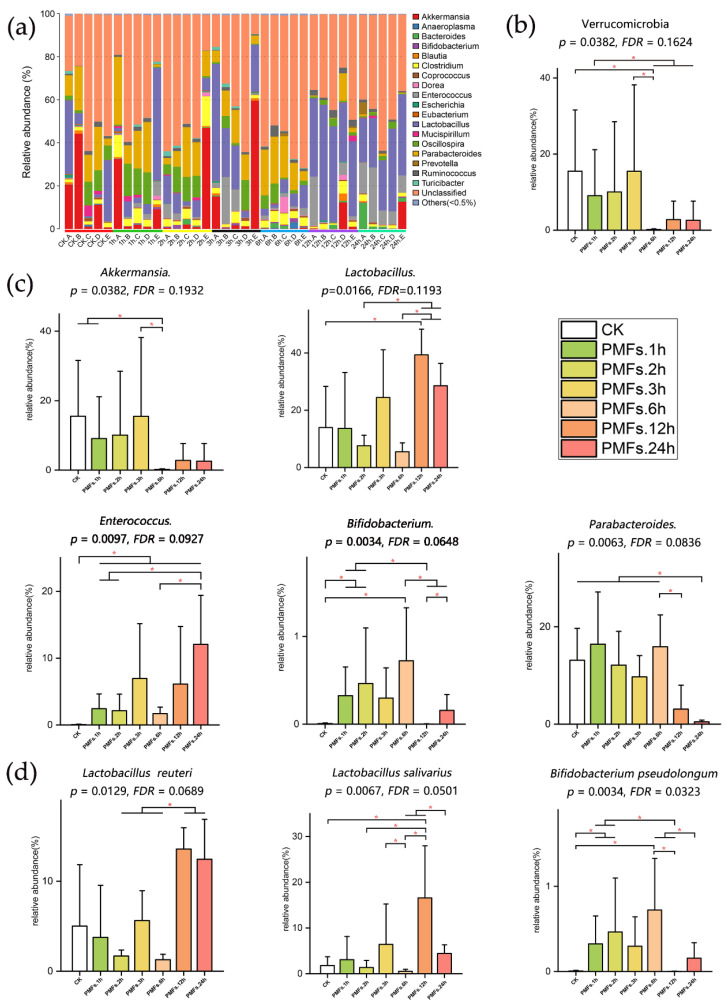
Specific effect of PMF-rich fraction on the composition and abundance of microbial communities. (**a**) The taxonomic composition distribution (genus level). “Others (<0.5%)” includes all the taxonomic groups with relative abundance less than 0.5%. (**b**) Effects of PMF-rich fraction on the composition of gut microbiota at the phylum level. (**c**) Effects of PMF-rich fraction on the composition of gut microbiota at the genus level. (**d**) Effects of PMF-rich fraction on the composition of gut microbiota at the species level. Data were expressed as mean ± SD. Kruskal–Wallis test was used for multi-group comparations, wherein the significance level was 0.05 and the *p*-value was adjusted in the false discovery rate (FDR) method. Wilcoxon rank-sum test was used for two group comparations. * *p* < 0.05, *n* = 5 individuals per group.

In order to assess the specific effect of PMF-rich fraction on the composition and abundance of microbial communities, we performed species annotation on the basis of OTU at the phylum, genus, and species levels. The distribution of taxonomic composition histograms of each sample at different levels are shown in Figure 3a and Figure S3. We further chose species with high relative abundance of each taxonomic rank to perform difference analysis, and the results are displayed in Figure 3 and Figure S4.

At the phylum level (Figure 3b and Figure S4a), no significant difference was observed in the composition of Firmicutes (*p* = 0.5237) and Bacteroidetes (*p* = 0.2403) among groups, whereas the relative abundance of Verrucomicrobia (*p* = 0.04, Figure 3b) was significantly altered. Compared with the control group, the relative abundance of Verrucomicrobia was significantly decreased in PMFs.6h (*p* = 0.0122).

At the genus level (Figure 3c and Figure S4b), no significant shift was observed in the compositions of *Oscillospira* (*p* = 0.4076) and *Blautia* (*p* = 0.3281), while the compositions of *Akkermansia* (*p* = 0.0382), *Lactobacillus* (*p* = 0.0166), *Parabacteroides* (*p* = 0.0063), *Bifidobacterium* (*p* = 0.0034), and *Enterococcus* (*p* = 0.0097) were significantly changed. The relative abundance of *Akkermansia* in group PMFs.6h was significantly decreased compared to the control group (*p* = 0.0122), PMFs.1h (*p* = 0.01219), and PMFs.3h (*p* = 0.0367). The relative abundance of *Lactobacillus* in group PMFs.12h was significantly increased compared to the control group (*p* = 0.0367), PMFs.2h (*p* = 0.0122), and PMFs.6h (*p* = 0.0122). As for *Parabacteroides*, the relative abundance of this species in PMFs.24h was significantly decreased compared to the control group, PMFs.1h, PMFs.2h, PMFs.3h, and PMFs.6h (*p* = 0.0122). Further, the relative abundance of *Enterococcus* was significantly increased after the administration of PMF-rich fraction at all time points when it was compared to the control group (*p* < 0.05). Moreover, compared to the control group, the relative abundance of *Bifidobacterium* in PMFs.1h (*p* = 0.0112), PMFs.2h (*p* = 0.0345), and PMFs.6h (*p* = 0.0112) was significantly increased. However, the relative abundance of *Bifidobacterium* was significantly decreased in PMFs.12h (*p* = 0.0367) and PMFs.24h (*p* = 0.0345) compared to PMFs.6h.

Further analysis at the species level (Figure 3d) revealed that the species that significantly changed in the genus *Lactobacillus* were *Lactobacillus reuteri* (*p* = 0.0129) and *Lactobacillus salivarius* (*p* = 0.0067), and that significantly changed in the genus *Bifidobacterium* was *Bifidobacterium pseudolongum* (*p* = 0.0034).

## 4. Discussion

Several studies have been carried out to investigate the metabolism of PMFs in vivo. Nevertheless, urine, feces, and blood plasma of animals were used in the majority of these studies, with only few studies measuring the metabolites in the intestinal contents. Goncalves et al. [27] identified seven metabolites in rat urine after the administration of PMFs, including tangeretin-4′-glucuronide, 3′,4′-dihydroxy-5,6,7-trimethoxy-4′-glucuronide, 4′-demethyltangeretin, 3′-demethylsinensetin, nobiletin-*O*-glucuronide, 4′-demethylnobiletin, and 3′,4′-dihydroxy-3,5,6,7,8-pentamethoxyflavone. Zheng et al. [28] found seven metabolites in mice urine after oral administration of nobiletin, which were 3′-demethylnobiletin, 4′-demethylnobiletin, 5-demethylnobiletin, 5,3′-demethylnobiletin, 5,4′-demethylnobiletin, 3′,4′-demethylnobiltin, and 5,3′,4′,-demethylnobiletin. Similar results were obtained in the study by Li et al. [29]. They found that 4′-demethylnobiletin was the main metabolite of nobiletin in mice urine, while there were also small amounts of 3′-demethylnobiletin and other dimethyl products. They also reported that the metabolites further underwent glucuronidation, sulfation, and demethylation. Zeng et al. [30] proposed that the metabolic pathway of nobiletin and tangeretin was systematically highly similar; the parental compounds underwent demethlylation, hydroxylation, and demethoxylation as the first step, and the metabolites generated in the first step further underwent glucuronidation.

In our study, intestinal content was used to investigate the metabolism of PMFs. A total of 21 metabolites of PMFs were identified. It is likely that these metabolites were generated mostly through demethylation, hydroxylation, demethoxylation, and glucuronidation. Our result agreed with the previous literature to some extent [27,28,29,30]. However, due to the co-existence of multiple isomers and the trace amount of some metabolites, the chemical structures of these metabolites are required to be further confirmed. Moreover, the high structural similarity of the parental compounds added another layer of barriers to identify the metabolic pathway of PMFs. It is possible that the same metabolites are derived from different parental compounds. Further research is required to match the metabolites with corresponding parental compounds by administrating individual parental compounds. It also needs to be confirmed as to whether a certain metabolite is generated through one-step-reactions or chain reactions. We speculated that the gut microbiota played a crucial role in metabolism of PMFs. However, the enzymes present in intestinal enterocytes may also be involved in the metabolic process. In addition, the metabolism of PMFs is likely to be influenced by the enterohepatic circulation. Germ-free or pseudo-germ-free animal models are needed to study the specific role of the gut microbiota on the metabolism of PMFs.

Our results showed that the administration of Ougan PMFs altered the gut microbiota composition. The administration of PMFs significantly increased the richness of the microbial community. However, no significant change was found in the diversity of the microbial community. Different analyses between different time points were performed to predict the trend of the microbial changes at different taxonomic levels. At the phylum level, the relative abundance of Verrucomicrobia was significantly decreased after 6 h oral administration of PMFs. At the genus level, a significant decrease in the composition of *Parabacteroides* was observed 24 h after PMF administration, while that of *Akkermansia* was significantly decreased 6 h after consuming PMFs. A significant increase was found in the composition of *Enterococcus*. The increase began 1 h after oral PMFs and its effect lasted at least until 24 h. Moreover, PMFs also caused a significant increase in the relative abundance of *Lactobacillus* 12 h later, and that of *Bifidobacterium* was found to significantly increase 6 h later. Further analysis on the species level revealed that *Lactobacillus reuteri* and *Lactobacillus salivarius* in *Lactobacillus*, and *Bifidobacterium pseudolongum* in *Bifidobacterium*, were significantly increased. Accordingly, it was as early as 1 h after oral PMFs that the gut bacteria were altered, and a more comprehensive spectrum of the changes were observed in 24 h.

There are only few studies investigating the influence of PMFs on gut microbiota. A recent study carried out by Tung et al. [31] examined the effects of citrus peel extracts on the gut microbiota of high-fat diet-induced obesity in C57BL/6 mice. Their results indicated that PMFs in citrus peel extracts were able to reverse the unbalanced gut microbiota caused by a high fat diet, mainly by increasing the abundance of *Prevotella* and decreasing the abundance of *rc4-4*. The differences between the results of this study and our current study can be caused by the mouse species, pathological states of mice, composition of PMFs, and treatment duration. Nevertheless, both studies indicated the potential health-promoting property of citrus PMFs through beneficially altering the composition of gut microbiota. A more recent study carried out by Man Zhang et al. [32] investigated the effects of aged citrus peel (*chenpi*) extract, which was rich in PMFs on colonic microbiota in high-fat diet induced obese mice. The results showed that after 11 weeks’ treatment, *chenpi* extract demonstrated a prebiotic effect, evidenced by the promoted *Lactobacillus* and *Bifidobacterium*. Their results were consistent with ours, which indicated that citrus PMFs could exert a prebiotic effect, both in a short-term and long-term manner. Moreover, their study also demonstrated the dynamics of *Akkermansia* in a dose- and time-dependent manner. However, in our study, the relative abundance of *Akkermansia* was found to be significantly decreased 6h after consuming PMFs, and it was not time-dependent. The difference of the changes in *Akkermansia* may be because of that 24 h was too short for *Akkermansia* to present a time-dependent manner in the present study.

The Food and Agriculture Organization of the United Nations has defined probiotics as live microorganisms that are able to promote human health when administrated in adequate amounts [33]. Bacteria classified in the genera *Lactobacillus* and *Bifidobacterium* are universally acknowledged as important probiotics [34]. They are butyrate-producing bacteria, and mounting studies have shown that they play significant roles in regulating and preventing diseases [35,36]. An et al. [37] indicated that some species of *Bifidobacterium* are related to fat loss, which could be utilized as a potential method for weight loss in obese individuals. Tuohy et al. [38] drew a conclusion that some species of *Lactobacillus* and *Bifidobacterium* reduced intestinal absorption of cholesterol, thus lowering the risks of cardiovascular disease. Yunes et al. [39] indicated that various species of *Lactobacillus* and *Bifidobacterium* produce neurotransmitter γ-gamma-amino butyric acid (GABA), which is beneficial in regulating different physiological activities after passing through the gut–brain axis. Hsu et al. [40] found that the supplement of *Lactobacillus reuteri* mitigated hepatic inflammation and apoptosis. In our current study, the supplement of Ougan PMFs significantly increased the relative abundances of *Lactobacillus* and *Bifidobacterium* in gut microbiota. *Lactobacillus* and *Bifidobacterium* were positively correlated with body health, and thus it could be implied that the citrus PMFs were able to exert health-promoting and disease-preventing ability, partly through beneficially regulating the gut microbiota.

**Figure 4:**
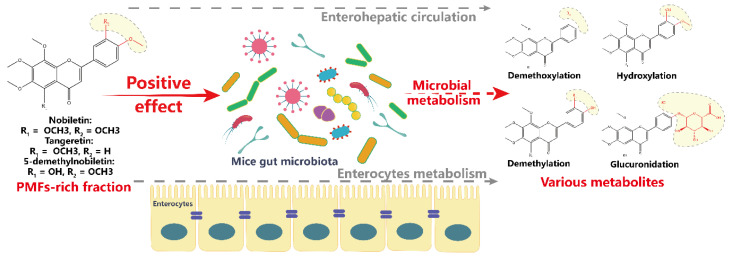
Hypothetical scheme of interactions between Ougan PMFs and the gut microbiota.

On the basis of the obtained results, we believe that there were possible interactions between Ougan PMFs and gut microbiota. In the intestinal contents, the main components of Ougan PMFs, nobiletin, tangeretin, and 5-demethylnobiletin, were metabolized and various metabolites were generated. These metabolites were mainly generated through demethylation, demethoxylation, hydroxylation, and glucuronidation. Although the metabolites may be generated through many metabolic pathways, such as enterocyte cell metabolism, enterohepatic circulation, and microbial metabolism, we speculated that the gut microbiota played the most important role. In turn, the administration of PMFs significantly altered the composition of gut microbiota, especially *Akkermansia*, *Lactobacillus*, *Parabacteroides*, *Bifidobacterium*, and *Enterococcus* at the genus level. The proposed interactions between Ougan PMFs and gut microbiota were shown in Figure 4. Further investigations using antibiotics or germ-free animals that feed Ougan PMFs are needed to confirm if gut microbiota are required to metabolize Ougan PMFs.

## 5. Conclusions

In conclusion, PMF-rich fraction extracted from Ougan fruit, with nobiletin, tangeretin, and 5-demethylnobiletin as main components, greatly altered the gut microbiota composition of mice. Two important probiotics, *Lactobacillus* and *Bifidobacterium*, were significantly increased after PMF administration, indicating a beneficial health-promoting effect of citrus PMFs through beneficially regulating the gut microbiota. Various metabolites of PMFs were detected and were generated through demethylation, hydroxylation, demethoxylation, and glucuronidation in the mouse gastrointestinal tract. However, there are still some limitations of the present study. Due to the lack of a single administration of each compound of the PMF-rich fraction, we found it difficult to match the metabolites with corresponding parental compounds. Moreover, it also caused hurdles for us to understand if all the single parental compounds had the same regulatory effects on the gut microbiota. Our study provided strong rationale for using PMFs as a food supplement to enhance human health, which warrants testing in the settings of clinical trials.

## Figures and Tables

**Table 1:** Flavonoids identification in Ougan polymethoxyflavone (PMF)-rich fraction by HPLC–ESI–MS/MS.

Peak No.	Retention Time (min)	[M + H]^+^ or [M − H]^−^ (*m*/*z*)	Error (ppm)	Formula	Fragment Ions (*m*/*z*)	Tentative Compounds
1	13.4438	609.1827 (-)	0.5	C_28_H_34_O_15_	489, 343, 301, 286, 242	Neohesperidin
2	21.2998	593.1876 (-)	0.0	C_28_H_34_O_14_	285	Poncirin
3	29.1432	373.1291	2.2	C_20_H_20_O_7_	358, 343, 327, 315, 299, 181, 163, 153	Isosinensetin
4	29.3402	389.1231	2.6	C_20_H_20_O_8_	374, 359, 344, 331, 313, 298, 287, 211, 183	Monohydroxy-pentamethoxyflavone
5	31.3811	373.1282	0.9	C_20_H_20_O_7_	357, 343, 329, 312, 297, 153	Sinensetin
6	31.8459	343.1176	0.8	C_19_H_18_O_6_	328, 313, 285, 257, 181, 153	Tetramethyl-*O*-isoscutellarein
7	33.4704	403.1387	0.6	C_21_H_22_O_8_	388, 373, 358, 355, 327, 211, 183	Nobiletin
8	33.853	375.1074	1.7	C_19_H_18_O_8_	360, 345, 330, 327, 317, 302, 197, 169, 149	5,4′-Dihydroxyl-3,7,8,3′-tetramethoxyflavone
9	34.0789	343.1176	2.0	C_19_H_18_O_6_	328, 313, 285, 257, 181, 153	Tetramethyl-*O*-scutellarein
10	35.9401	373.1282	0.3	C_20_H_20_O_7_	358, 343, 328, 297, 211, 183	Tangeretin
11	37.8647	389.1231	0.5	C_20_H_20_O_8_	374, 359, 341, 331, 197	5-demethylnobiletin

**Table 2:** Identification of intestinal metabolites of Ougan PMF-rich fraction by HPLC–ESI–MS/MS.

Peak No.	Retention Time (min)	[M + H]^+^ or [M − H]^−^ (*m*/*z*)	Formula	Fragment Ions (*m*/*z*)	Tentative Compounds
1	13.1284	609.1825 (-)	C_28_H_34_O_15_	489, 343, 325, 301, 286, 257, 242, 164	Neohesperidin
2	16.3972	563.1460 (-)	C_26_H_28_O_14_	387, 372, 357, 342, 314, 299, 175, 113	Nobelitin-*O*-glucuronide (1)
3	18.8731	535.1449	C_25_H_26_O_13_	359, 344, 329, 314, 311, 301, 298, 286, 283, 257	Tangeretin-*O*-glucuronide (1)
4	19.5825	565.1555	C_26_H_28_O_14_	389, 374, 359, 345, 344, 343, 341, 331, 328, 327, 316, 315, 313	Nobelitin-*O*-glucuronide (2)
5	19.8712	565.1551	C_26_H_28_O_14_	389, 374, 359, 345, 344, 343, 341, 339, 331, 329, 328, 327, 316, 315, 313, 310, 301, 298	Nobelitin-*O*-glucuronide (3)
6	20.1621	375.1078	C_19_H_18_O_8_	360, 345, 330, 327, 317, 302, 299, 197, 169, 149	Dihydroxy-tetramethoxyflavone (1)
7	21.0102	287.0917	C_16_H_14_O_5_	161, 153, 135, 133, 125, 121, 118, 111, 103, 97, 69, 67	Dihydroxy-monomethoxyflavone
8	21.5526	345.0974	C_18_H_16_O_7_	330, 315, 297, 287, 272, 197, 169	Dihydroxy-trimethoxyflavone
9	22.4321	375.1081	C_19_H_18_O_8_	360, 345, 330, 327, 317, 302, 197, 169	Dihydroxy-tetramethoxyflavone (2)
10	22.6898	327.0874 (-)	C_18_H_16_O_6_	327, 312, 297, 282, 269, 254, 226, 182, 177, 117	Monohydroxy-trimethoxyflavone
11	23.5766	535.1449	C_25_H_26_O_13_	359, 344, 329, 311	Tangeretin-*O*-glucuronide(2)
12	24.0618	375.1081	C_19_H_18_O_8_	360, 345, 331, 330, 327, 317, 314, 302, 301, 299, 287, 285, 274, 273, 271, 211, 183, 168, 165, 147, 139, 137, 135, 134, 127	Dihydroxy-tetramethoxyflavone (3)
13	24.2942	375.1084	C_19_H_18_O_8_	360, 345, 331, 330, 327, 325, 317, 314, 313, 302, 299, 287, 285, 274, 273, 271, 230, 211, 183, 168, 137, 135, 127	Dihydroxy-tetramethoxyflavone (4)
14	24.6514	565.1555	C_26_H_28_O_14_	389, 374, 359, 356, 341	Nobelitin-*O*-glucuronide (4)
15	25.8876	389.1237	C_20_H_20_O_8_	374, 359, 356, 341, 331, 316, 285, 244, 197, 169, 163, 148, 113	Monohydroxy-pentamethoxyflavone (1)
16	26.0809	271.0607	C_15_H_10_O_5_	253, 243, 215, 197, 169, 153, 149, 115, 91	Trihydroxy-flavone
17	26.6764	359.1135	C_19_H_18_O_7_	344, 343, 341, 329, 327, 325, 315, 301, 300, 298, 297, 283, 272, 255, 227, 181, 153	Monohydroxy-tetramethoxyflavone (1)
18	27.7937	389.1238	C_20_H_20_O_8_	374, 359, 344, 343, 341, 331, 316, 197, 169, 165, 163	Monohydroxy-pentamethoxyflavone (2)
19	28.4142	359.1128	C_19_H_18_O_7_	344, 329, 315, 314, 311, 309, 301, 298, 297, 286, 285, 283, 271, 268, 258, 257, 255, 240, 230, 228, 215, 214, 212, 211, 200, 193, 187, 183, 168, 165, 139, 131, 127, 121	Monohydroxy-tetramethoxyflavone (2)
20	29.1096	389.1234	C_20_H_20_O_8_	374, 359, 345, 344, 343, 341, 339, 331, 328, 326, 316, 315, 313, 310, 301, 299, 288, 287, 285, 270, 260, 245, 230, 217, 211, 193, 183, 168, 151, 127	Monohydroxy-pentamethoxyflavone (3)
21	29.5447	359.0772	C_17_H_14_O_6_	344, 329, 314, 311, 301, 286, 283, 258, 242, 230, 214, 202, 193, 177, 174, 133	Dihydroxy-dimethoxyflavone
22	30.7912	359.1135	C_19_H_18_O_7_	344, 329, 314, 311, 301, 286, 283, 197, 169, 133	Monohydroxy-tetramethoxyflavone (3)
23	31.4917	343.1182	C_19_H_18_O_6_	328, 313, 285, 181, 153, 133	Tetramethoxyflavone
24	33.2004	403.1395	C_21_H_22_O_8_	388, 387, 373, 359, 358, 357, 355, 353, 345, 343, 341, 339, 330, 327, 325, 315, 313, 311, 301, 299, 259, 257, 244, 231, 211, 193, 183, 175, 168, 163, 162	Nobiletin
25	35.6699	373.1291	C_20_H_20_O_7_	358, 343, 325, 315, 300, 297, 283, 271, 269, 229, 211, 193, 183, 168, 135	Tangeretin
26	37.6439	389.1241	C_20_H_20_O_8_	374, 359, 356, 341, 343, 328, 316, 313, 197, 169, 163	5-demethylnobiletin

## Supplementary Materials

The following are available online at https://www.mdpi.com/2076-3921/9/9/831/s1, Table S1: PCR conditions. Figure S1: Alpha diversity indices boxplot among groups. Figure S2: Log-scaled percentage heat map based on the relative abundance of each species in each sample (species level). Figure S3: The taxonomic composition distribution. Figure S4: Effects of PMF-rich fraction on the composition of gut microbiota.
