# Supplementary material for: Beneficial Regulatory Effects of Polymethoxyflavone—Rich Fraction from Ougan (Citrus reticulata cv. Suavissima) Fruit on Gut Microbiota and Identification of Its Intestinal Metabolites in Mice
Source: Antioxidants (Basel). 2020 Sep 6;9(9):831. doi: 10.3390/antiox9090831 (PMC7555910; doi:10.3390/antiox9090831)
Supplement: Supplementary file 1 [file antioxidants-09-00831-s001.pdf]

Supplementary Materials

Table S1. PCR conditions

Both forward and reverse primers were tagged with Illumina adapter, pad and linker sequences. PCR enrichment was performed in a 50 μL reaction containing 30ng template, fusion PCR primer and PCR master mix. PCR cycling conditions were as follows: 95 °C for 3 minutes, 30 cycles of 95 °C for 45 seconds, 56 °C for 45 seconds, 72 °C for 45 seconds and final extension for 10 minutes at 72 °C for 10 minutes. The PCR products were purified using Agencourt AMPure XP beads and eluted in Elution buffer. Libraries were qualified by the Agilent Technologies 2100 bioanalyzer. The validated libraries were used for sequencing on Illumina HiSeq 2500 platform (BGI, Shenzhen, China) following the standard pipelines of Illumina, and generating 2 × 250 bp paired-end reads.

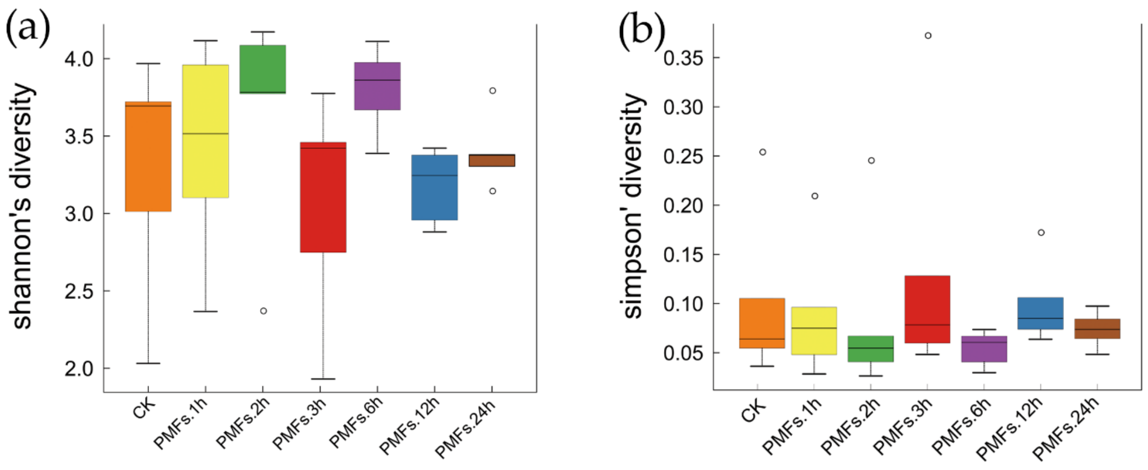

Figure S1. Alpha diversity indices boxplot among groups. (**a**) Shannon diversity index; (**b**)Simpson diversity index. Five lines from bottom to top is the minimum value, the first quartile, median, the third quartile and the maximum value, and the outliers are shown as 'o'. * $p < 0.05$.

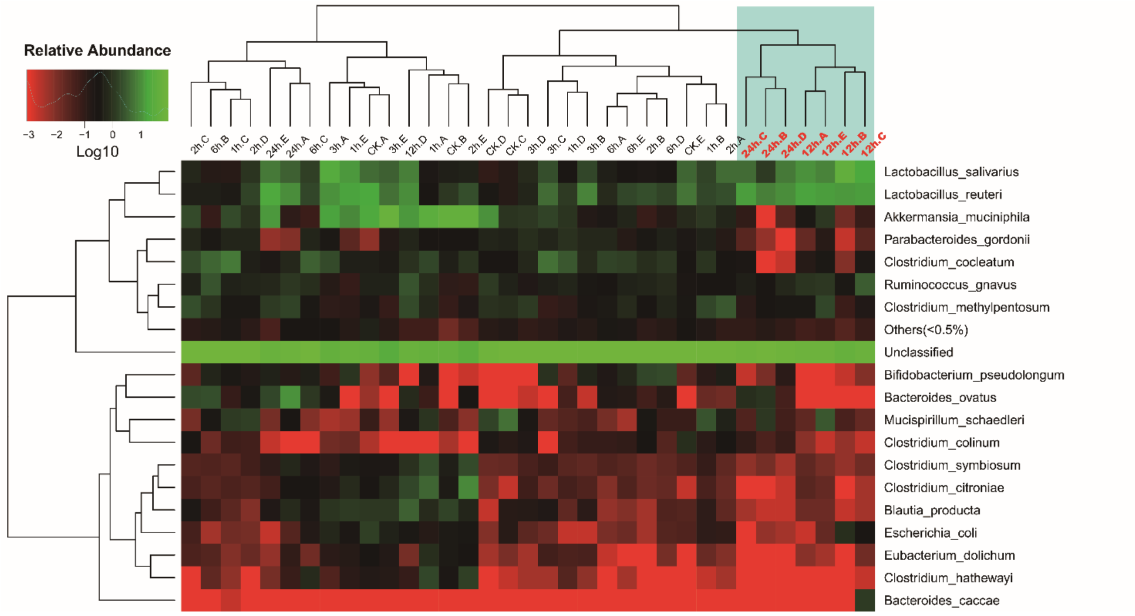

Figure S 2. Log-scaled percentage heat map based on the relative abundance of each species in each sample (Species level). Longitudinal clustering indicates the similarity of all species among different samples, and the horizontal clustering indicates the similarity of certain species among different samples. The closer the distance, the shorter the branch length, and the more similar the species composition between the samples. Relative abundance values were all log transformed. Most of the samples of groups PMFs.12h and PMFs.24h were clustered into the same group, highlighted in blue in right-top corner.

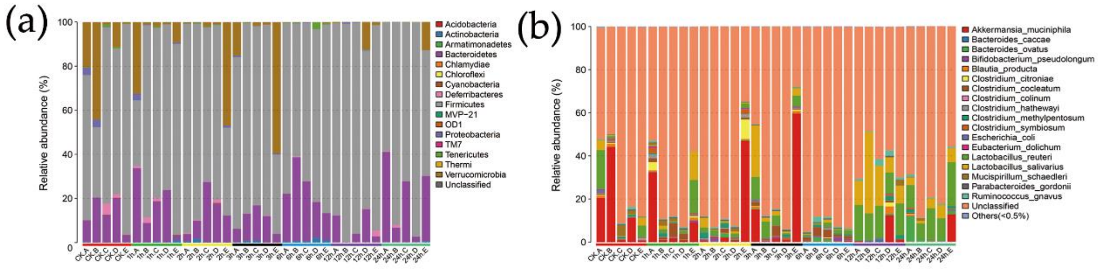

Figure S3. The taxonomic composition distribution. (**a**) Phylum level; (**b**) Species level. "Others (< 0.5%)" includes all the taxonomic groups with relative abundance less than 0.5%.

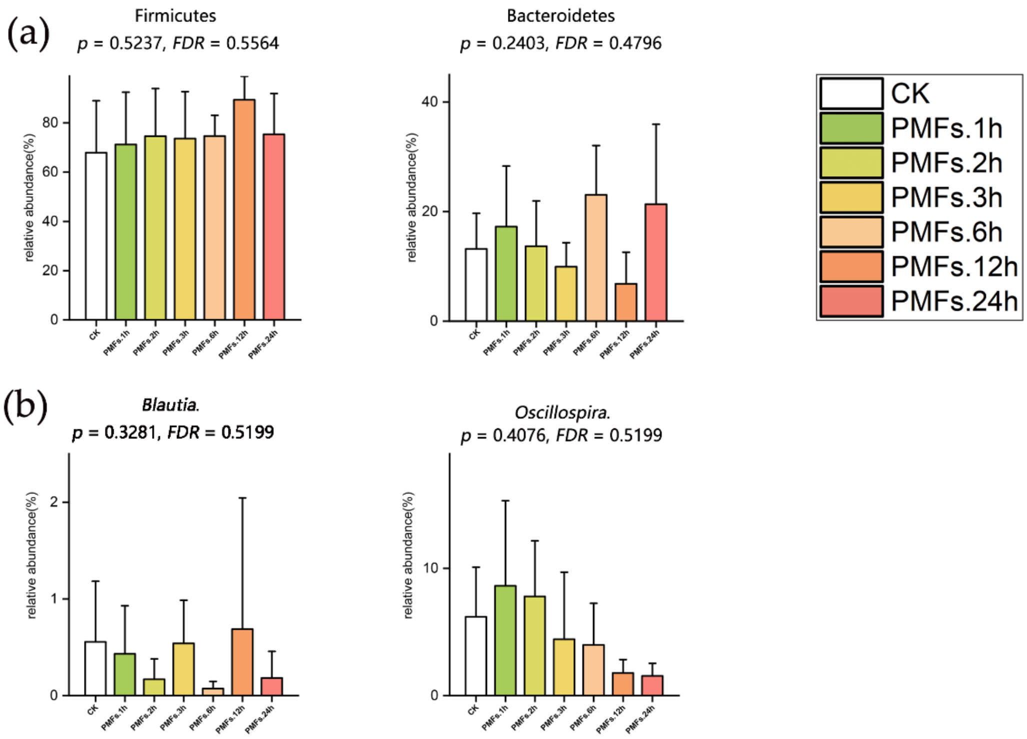

Figure S4. Effects of PMFs-rich fraction on the composition of gut microbiota. (**a**) Phylum level; (**b**) Genus level. Data were expressed as mean ± SD. Kruskal-Wallis Test was used for multi-group comparations, significant level was 0.05, *p* value was adjusted in the false discovery rate (FDR) method. Wilcoxon Rank-Sum Test is used for two groups comparations.
